# The Impact of COVID-19 on Adolescent Mental Health: Preliminary Findings From a Longitudinal Sample of Healthy and At-Risk Adolescents

**DOI:** 10.3389/fped.2021.622608

**Published:** 2021-06-08

**Authors:** Zsofia P. Cohen, Kelly T. Cosgrove, Danielle C. DeVille, Elisabeth Akeman, Manpreet K. Singh, Evan White, Jennifer L. Stewart, Robin L. Aupperle, Martin P. Paulus, Namik Kirlic

**Affiliations:** ^1^Laureate Institute for Brain Research, Tulsa, OK, United States; ^2^Department of Psychology, University of Tulsa, Tulsa, OK, United States; ^3^Department of Psychiatry and Behavioral Sciences, Stanford University, Palo Alto, CA, United States; ^4^School of Community Medicine, University of Tulsa, Tulsa, OK, United States

**Keywords:** adolescents, anxiety, depression, COVID-19, stress

## Abstract

**Background:** The COVID-19 pandemic has brought on far-reaching consequences for adolescents. Adolescents with early life stress (ELS) may be at particular risk. We sought to examine how COVID-19 impacted psychological functioning in a sample of healthy and ELS-exposed adolescents during the pandemic.

**Methods:** A total of 24 adolescents (15 healthy, nine ELS) completed self-report measures prior to and during the COVID-19 pandemic. The effect of COVID-19 on symptoms of depression and anxiety were explored using linear mixed-effect analyses.

**Results:** With the onset of the pandemic, healthy but not ELS-exposed adolescents evidenced increased symptoms of depression and anxiety (*p*s < 0.05). Coping by talking with friends and prioritizing sleep had a protective effect against anxiety for healthy adolescents (*t* = −3.76, *p* = 0.002).

**Conclusions:** On average, this study demonstrated large increases in depression and anxiety in adolescents who were healthy prior to the COVID-19 pandemic, while ELS-exposed adolescents evidenced high but stable symptoms over time.

## Introduction

Beyond the physical health consequences of the virus (1), the COVID-19 pandemic is expected to have a substantial impact on mental health (2), particularly for adolescents and young adults (3). Although COVID-19 does not place most adolescents at significant physical risk (4, 5), the burgeoning literature suggests that many aspects of the global pandemic (e.g., fear of infection, social disconnectedness, and financial difficulties) increase stress reactions and pose a threat to mental health in adolescents (6). This is further compounded by already rising rates of internalizing disorders in adolescents and young adults and seeking treatment for psychopathology (7–9). Due to the importance of peer relationships in adolescence (6, 10), consequences of the pandemic measures, such as school closures and lack of social contact, may be especially difficult for this population.

Early life stress (ELS; e.g., child abuse and neglect, domestic violence, and parental psychopathology) is a salient risk factor that may predispose individuals to negative outcomes from large scale stressors such as COVID-19 (11). Adolescents who have experienced ELS are more likely to develop both internalizing (12) and externalizing disorders (13). ELS-exposed adolescents demonstrate increased threat reactivity (14), experience difficulties in emotion regulation (15), and tend to use maladaptive coping skills [e.g., avoidance, substance use, risk-taking behaviors; (16)], which may place them at higher risk for experiencing negative mental health consequences of COVID-19 relative to adolescents without ELS histories.

Although other studies have examined the impact of COVID-19 on mental health symptoms in adolescents (5)^1^, none of the prior investigations compare these effects against pre-pandemic measures. Because the pandemic presented unique challenges and stressors that could negatively impact the well-being of adolescents, research is needed to determine how adolescents' mental health has been affected and whether those already at risk are disproportionally affected. Therefore, the present study aimed to (1) describe adolescent experiences during the COVID-19 pandemic, (2) examine the impact of the pandemic on mental health symptoms relative to pre-pandemic functioning in healthy and adolescents with ELS exposure, and (3) explore what factors may account for changes in symptoms. The study was conducted at a private research institute in the larger Tulsa, Oklahoma, metro area. Adolescents were surveyed at two timepoints: (1) prior to the onset of COVID-19 (1–8 months prior to the pandemic) as part of a larger longitudinal study and (2) ~3 months after the first COVID-19 case was identified in Oklahoma. Because the existing literature suggests that COVID-19 poses a threat to mental health, especially for adolescents, we predicted that adolescents would show an overall trend toward increased mental health problems following the onset of the pandemic. We further expected that this trend would be greater for adolescents with ELS exposure than for healthy controls (HC). Finally, we conducted a series of exploratory analyses to identify potential risk and resilience factors (e.g., social functioning, coping skills, family dynamics) for changes in mental health symptomatology.

## Methods

### Participants

Twenty-four adolescents [15 healthy control [HC] and nine with early life stress exposure [ELS]] participated in the present study as part of a larger longitudinal investigation of (1) the development and maintenance of mood, anxiety, and stress disorders in adolescents [Neuroscience-Based Mental Health Assessment and Prediction for Adolescents (NeuroMAP-A)] and (2) how mindfulness training augmented with real-time functional magnetic resonance imaging (rtfMRI-nf) may enhance resilience in adolescents [Augmented Mindfulness Training for Resilience in Early Life (A-MindREaL)], both funded by the National Institute for General Medical Sciences Centers of Biomedical Research Excellence (CoBRE) grant. Participants were recruited from the community using a variety of methods, including a school messaging platform (PeachJar), radio advertisements and billboards, social media posts, news broadcasting, and word of mouth.

At the time of data collection, a total of 29 adolescents (17 HC, 12 ELS) completed the baseline visits (August 2019–February 2020) for this longitudinal investigation. All subjects were invited to complete follow-up questionnaires for the present study. Five of the 29 participants either could not be reached for follow-up or declined to participate, resulting in a total sample of 24 adolescents. HC adolescents were psychiatrically healthy and reported no history of maltreatment. ELS participants reported histories of childhood maltreatment and met criteria for at least one anxiety and/or depressive disorder, assessed by the Mini International Neuropsychiatric Interview for Children and Adolescents (17). Parents provided written informed consent, while adolescents provided assent for study participation. Parent consent and adolescent assent were both required for the baseline visit at study entry and in order to be sent questionnaires for this follow-up time point. All procedures were approved by the Western Institutional Review Board.

### Procedures

The Maltreatment and Abuse Chronology and Exposure Scale [MACEs; (18)] was used to assess exposure to maltreatment. Anxiety and depression were assessed using the pediatric Patient-Reported Outcomes Measurement Information System [PROMIS; (19)]. Family conflict was assessed using the Conflict subscale of the Family Environment Scale [FECS; (20)], while parent and peer relationship quality was assessed using the Inventory of Parent and Peer Attachment–Revised [IPPA-R; (21)]. Adolescents completed self-report assessments of ELS, mental health, and family and peer relationships at both timepoints. COVID-19-specific measures were administered following the onset of the pandemic (see below for timeline), including the COVID-19 Adolescent Symptom and Psychological Experience Questionnaire [CASPE; (22)], Adolescent Social Connectedness and Coping during COVID-19 Questionnaire [ASC; (23)], and Coronavirus Health Impact Survey [CRISIS; (24)].

#### COVID-19 Context

**Figure 1:**
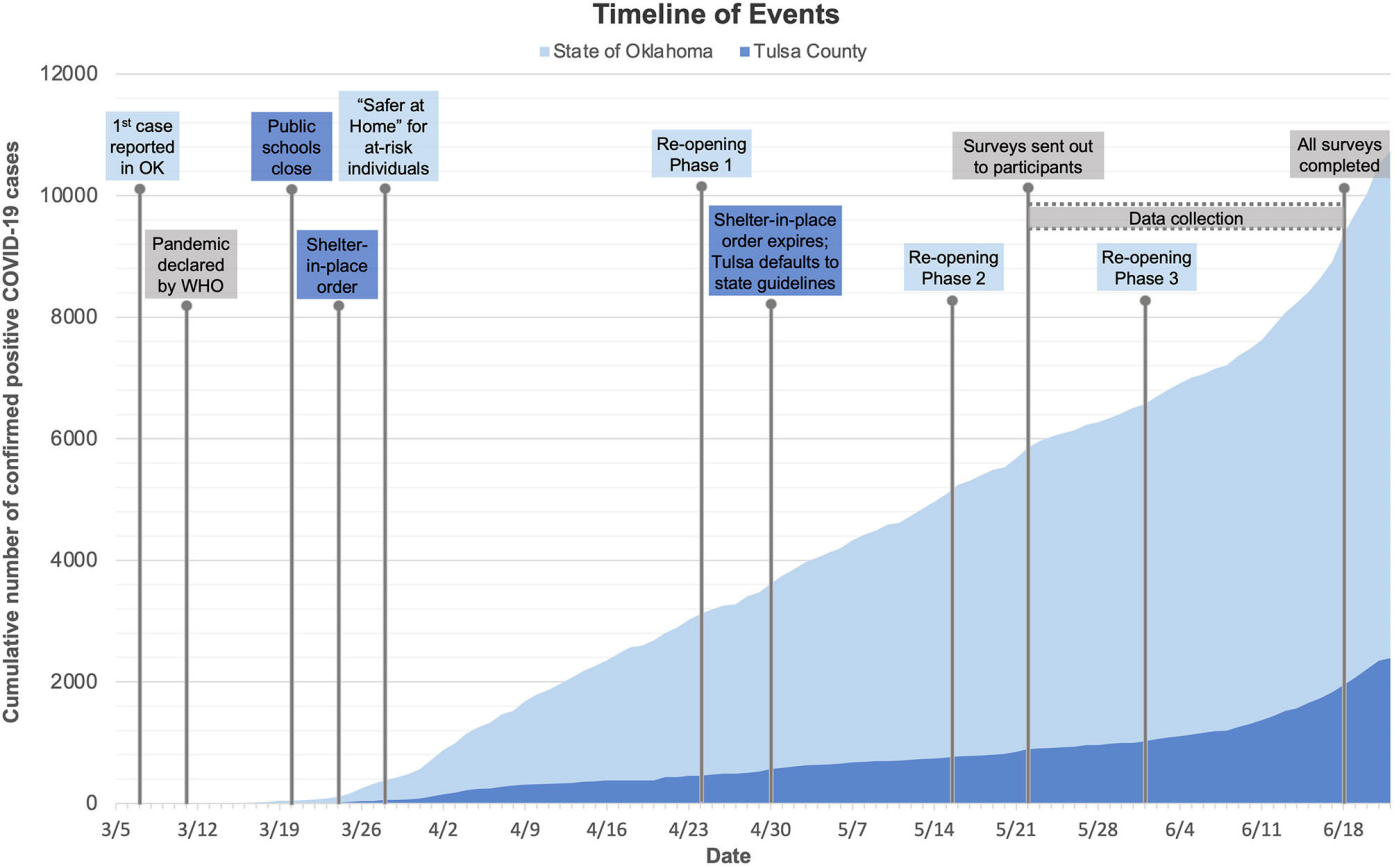
A timeline of state and local government restrictions and the trajectory of total cases. Between the pre-pandemic baseline and the COVID-19 follow-up assessment, participants had experienced the onset of the pandemic, a shelter-in-place order, rising rates of local cases, and a push toward reopening.

On March 7th, 2020, the first case of COVID-19 was announced in Oklahoma. By the 11th, the WHO had declared the disease a global pandemic (25). State and local responses to the pandemic came mid-month, with public schools announcing closures on March 20th, followed by state and city “Safer at Home” orders on the 24th and 28th, respectively (i.e., closure of non-essential businesses, making only essential trips outside of the household). While the state “Safer at Home” orders were limited to those 65 years or older and individuals with underlying conditions (26), the local orders encompassed all residents of Tulsa County. By March 31st, cases of COVID-19 in Oklahoma had increased to 565 and 23 deaths. Although cases and deaths continued to rise at both state and local levels, the state began a three-phased reopening plan on April 24th. On April 30th, Oklahoma reported a total of 3,618 positive cases and 222 deaths. Despite rising cases, the state continued a phased reopening in early May 2020. At the conclusion of the present study on June 18th, all restrictions were lifted by June 1st, as cases totaled 9,354 infections and 366 deaths. Surveys for the current study were sent out on May 22nd and completed by June 18th. Thus, 23 adolescents provided follow-up responses during the period overlapping with Phase 2, while one adolescent provided responses during Phase 3 of the reopening plan, which began on May 15th and June 1st, respectively (26). Therefore, between the pre-pandemic baseline and the COVID-19 follow-up assessment, participants had experienced the onset of the pandemic, a shelter-in-place order, rising rates of local cases, and a push toward re-opening. See Figure 1 for a timeline for the follow-up period.

### Analysis

All statistical analyses were performed using R statistical package (27). Descriptive statistics regarding participant characteristics were obtained using the R package “psych” (28). Independent sample *t*-tests examined group differences on demographic variables. To examine changes in self-reported mental health symptoms after the onset of the pandemic, linear mixed-effects models (LMEs) were conducted using the “lmer” function in R package “lme4” (29) and plots were generated with “emmeans” (30) R package. Fixed effects included group (HC vs. ELS) and timepoint (pre-COVID-19 vs. COVID-19). Random effects included subject. Follow-up pairwise comparisons were conducted using estimated marginal means to further describe the effects of group and timepoint on the outcome variables.

Results were Bonferroni-corrected for multiple comparisons for primary outcome variables (depression, anxiety; a *p*-value cutoff of 0.05/1.6 = 0.03), secondary (family conflict, family and peer alienation, communication, and trust; a *p*-value cutoff of 0.05/5 = 0.01), and exploratory (correlations: *p*-value cutoff of 0.05/11 = 0.005; *t*-tests: *p*-value cutoff of 0.05/15 = 0.003). Given the small sample size, we report effect sizes calculated using Cohen's *d* for all outcomes and adjust our interpretations accordingly. A *post-hoc* exploration of the relationship between HC participants' change in anxiety and depression symptoms (i.e., calculated by subtracting the follow-up scores from the pre-COVID-19 scores, divided by the baseline score to produce change score independent of initial symptom severity) and a number of self-report measures related to the COVID-19 pandemic were examined using Spearman rank-order correlations or paired sample *t*-tests.

## Results

Between-group comparisons of demographics and clinical characteristics are presented in Table 1, while COVID-related experiences are presented in Supplementary Table 1. There were no significant differences between groups in the experience of COVID-19 symptoms, exposure, other pandemic-related impacts (e.g., family job/income loss, changes in routine), or coping skills (*p* > 0.05). Four adolescents (two ELS, two HC) reported physical symptoms of COVID-19, while two adolescents (one ELS, one HC) reported receiving a confirmed positive test result, and one adolescent (ELS) reported a suspected positive but was not tested. The majority of participants did not report knowledge of financial impacts related to COVID-19 and reported that at least one adult in the household was considered an essential worker. Notably, ELS participants reported experiencing greater negative emotions (*t* = 2.25, *p* = 0.03) and fewer positive emotions (*t* = −2.76, *p* = 0.01) than HC participants. Across both groups, it was noted that the majority began reengaging in activities within the community following the lifting of stay-at-home restrictions (e.g., contact with extended family, activities in public, family travel, eating in restaurants).

**Table 1:** Sample demographic and maltreatment exposure characteristics.

**Characteristic**	**ELS (*****n*** **= 9)**		**HC (*****n*** **= 15)**		**Group differences**
	**Mean**	**SD**	**Mean**	**SD**	***t***
Age	15.22	0.97	14.53	1.3	1.37
Grade in school	8.78	1.09	8.67	1.23	0.22
Income	86,777.78	74,264.36	102,066.67	80,293.63	−0.46
Psychotropic medication	0.33	0.5	0	0	2.62^**^
MACEs	16.89	5.9	3	3.09	7.6^***^
	***N***	**%**	***N***	**%**	**χ^2^**
Sex					3.7^**^
Male	1	11.1	9	60	
Female	8	88.9	6	40	
Ethnicity					0
Hispanic	1	11.1	2	13.3	
Non-Hispanic	8	88.9	13	86.6	
Race					0.64
White	6	67	9	60	
Native	0	0	2		
Mixed	3	33	4		

*** *p < 0.01*;

** *p < 0.05*;

* *p < 0.10*.

*ELS, early life stress; HC, healthy control; MACES, Maltreatment and Abuse Chronology and Exposure Scale*.

### Primary and Secondary Outcomes

**Figure 2:**
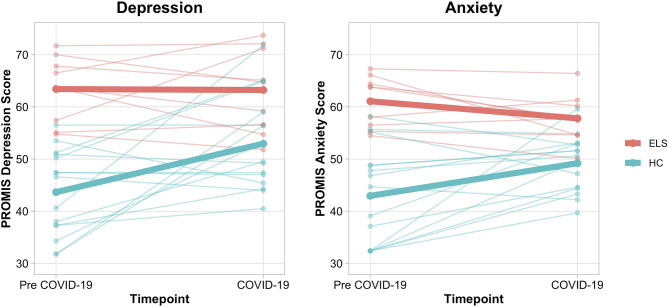
Spaghetti plots depicting self-reported depression and anxiety in healthy controls and early life stress exposed adolescents. Means for each group are depicted via a bolded line. A significant Group by Time Interaction was found for the LME models examining depression [*F*_(1, 22)_ = 4.54, *p* = 0.045, *d* = 0.91] and anxiety [*F*_(1, 22)_ = 9.35, *p* = 0.009, *d* = 1.23]. HC subjects demonstrated meaningful increases in depression and anxiety (*t* = 3.43, *p* = 0.003, *d* = 1.07; *t* = 3.10, *p* = 0.005, *d* = 0.81), while ELS subjects did not (*t* = −0.05, *p* = 0.96; *t* = −1.25, *p* = 0.222). ELS, early life stress; HC, healthy control; LME, Linear Mixed-Effects Model; PROMIS, Patient-Reported Outcomes Measurement System.

Table 2 details LME models results. For the LME examining depression, we observed a Group-by-Timepoint interaction [*F*_(1, 22)_ = 4.54, *p* = 0.045, *d* = 0.91; Figure 2]. Pairwise contrasts revealed that HCs exhibited significant increases in symptoms of depression (*t* = 3.43, *p* = 0.003, *d* = 1.07) following COVID-19 onset (46.7% reported an increase of 1+ SD), whereas the ELS group did not (*t* = −0.05, *p* = 0.96). Similar patterns were noted for anxiety. We observed a Group-by-Timepoint interaction [*F*_(1, 22)_ = 9.35, *p* = 0.009, *d* = 1.23] characterized by increased anxiety from pre-COVID-19 to COVID-19 timepoints among HCs (*t* = 3.10, *p* = 0.005, *d* = 0.81; 33.3% reported an increase of 1+ SD). Changes in anxiety were non-significant for ELS adolescents (*t* = −1.25, *p* = 0.222). Finally, there were no significant Group-by-Timepoint interactions to suggest that the experience of COVID-19 affected adolescents in the ELS or HC group disproportionally across family (i.e., family conflict) or peer domains (i.e., trust, communication, or alienation). In the absence of significant interactions, we examined main effects of Timepoint. We found that reports of peer trust [F_(1, 22)_ = 5.81, *p* = 0.025, *d* = 0.37] and peer communication [F_(1, 22)_ = 5.63, *p* = 0.027, *d* = −0.04] declined for both HC and ELS adolescents from the pre-COVID-19 to COVID-19 Timepoints, although no significant changes were observed for mothers' trust [F_(1, 22)_ = 0.01, *p* = 0.94, *d* = 0.06], fathers' trust [F_(1, 22)_ = 2.25, *p* = 0.15, *d* = 0.03], mothers' communication [F_(1, 22)_ = 0.32, *p* = 0.58, *d* = 0.15], or fathers' communication [F_(1, 22)_ = 0.15, *p* = 0.70, *d* = 0.08], or overall family conflict [F_(1, 22)_ = 0.96, *p* = 0.34, *d* = −0.60] for either HC or ELS adolescents.

**Table 2:** Unadjusted means, standard deviations, effect sizes, and main analyses of change from baseline in early life stress exposed compared with healthy controls.

**Outcome variable**	**Mean and standard deviation**				**Statistic**			
	**ELS**		**HC**		**Cohen's *d***	**Effect of COVID-19**	**SE**	***t***
**PROMIS**	**Random factor: subject**							
Depression								
Baseline	63.41	6.33	43.65	8.20				
Follow-up	63.23	8.07	52.95	9.13	0.91	9.47^**^	4.45	2.13
Anxiety								
Baseline	61.08	4.96	42.95	9.49				
Follow-up	57.80	4.64	49.20	5.46	1.23	9.53^***^	3.30	2.89
**Family conflict**	**Random factor: subject**							
Baseline	3.44	1.59	3.33	1.59				
Follow-up	4.22	1.48	3.20	1.32	−0.59	−0.91	0.66	−1.39
**Family attachment**	**Random factor: subject**							
Mom alienation								
Baseline	17.33	5.34	22.67	4.72				
Follow-up	16.89	5.06	21.80	3.91	−0.08	−0.42	2.29	−0.18
Dad alienation								
Baseline	13.25	7.96	21.67	5.83				
Follow-up	16.89	8.61	21.80	4.14	−0.70	−4.24	2.67	−1.59
Mom communication								
Baseline	25.33	8.12	30.87	10.09				
Follow-up	23.44	5.55	30.40	8.53	0.15	1.42	4.17	0.34
Dad communication								
Baseline	16.25	7.34	26.53	11.22				
Follow-up	16.11	11.22	27.60	8.54	0.08	0.72	3.65	0.20
Mom trustworthiness								
Baseline	33.56	9.38	39.67	8.90				
Follow-up	33.11	8.37	39.80	8.27	0.06	0.58	4.23	0.89
Dad trustworthiness								
Baseline	20.50	9.71	36.67	11.60				
Follow-up	22.67	8.00	39.73	7.36	0.03	0.31	3.88	0.08
**Peer attachment**	**Random factor: subject**							
Peer alienation								
Baseline	24.33	2.83	27.87	3.48				
Follow-up	21.67	5.39	26.53	3.46	0.27	1.33	2.08	0.64
Peer communication								
Baseline	34.33	5.02	34.33	5.74				
Follow-up	31.33	6.76	31.07	7.27	−0.04	−0.27	2.64	−0.10
Peer trustworthiness								
Baseline	44.78	4.09	47.27	5.15				
Follow-up	39.33	9.58	44.73	6.40	0.37	2.91	3.31	0.88

*** *p < 0.01*;

** *p < 0.05*;

* *p < 0.10*.

*ELS, early life stress; HC, healthy control; PROMIS, Patient-Reported Outcomes Measurement Information System*.

### Exploratory Outcomes

To contextualize the observed changes in depression and anxiety within the HC group, we conducted exploratory *post-hoc* analyses (Supplementary Tables 2, 3). We focused our analyses on strategies employed by 20–80% of the HC adolescents, correcting for multiple comparisons (*p* < 0.003). We found that increased depressive symptoms were correlated with reduced peer trust (*r*_*s*_ = −0.54, *p* = 0.036) during COVID-19. Further, increased anxiety was correlated with reduced peer trust (*r*_*s*_ = −0.64, *p* = 0.009) and increased hopelessness (*r*_*s*_ = 0.55, *p* = 0.032). Adolescents who endorsed video games as a coping strategy reported greater increases in depression (*t* = −4.18, *p* < 0.001, *d* = −0.45) and anxiety (*t* = −5.50, *p* < 0.001, *d* = −0.61) during COVID-19, whereas prioritizing good sleep (*t* = −3.97, *p* = −0.001, *d* = −0.39) and talking with friends virtually (*t* = −3.76, *p* = −0.002, *d* = −0.59) were associated with attenuated increases in anxiety during COVID-19.

## Discussion

The COVID-19 pandemic presented long-term challenges beyond physical health, affecting economic, social, and mental health domains (1). The pandemic may be particularly challenging for adolescent populations, especially those with history of early life stress exposure due to the increased mental health risk. To understand the impact of the pandemic on these populations, the present study assessed mental health symptoms and a variety of related factors (e.g., family dynamics, social functioning, and coping styles) in a sample of adolescents with and without ELS. Although prior studies have examined the association between adolescent mental health following COVID-19, to our knowledge there have been no longitudinal investigations of mental health in healthy and at-risk adolescents before and after the onset of COVID-19. We hypothesized that adolescents would demonstrate an increase in mental health symptoms with the onset of the pandemic and that this increase would be greater for those with ELS exposure.

Our hypothesis for symptom changes across groups was not supported. On average, healthy adolescents exhibited large increases in self-reported anxiety and depression, while symptoms in adolescents with ELS remained high yet stable following the pandemic's onset. Indeed, nine of 15 healthy adolescents exhibited clinically meaningful increases in self-reported anxiety and depression, while the ELS group's reported symptoms did not change following the onset of COVID-19. It is possible that ELS adolescents' perception of stress remained the same during the pandemic, given their chronic stress exposure. Additionally, ELS adolescents may have accessed already-established internal or external resources, including connection to psychotherapy services (two-thirds of ELS adolescents) or use of prescribed selective serotonin reuptake inhibitors (one-third). On average, healthy adolescents denied using these resources, including two adolescents who reported starting mental health professional counseling, while none reported starting new prescription medications.

Our findings suggest that COVID-19 related changes in mental health symptoms in healthy adolescents were associated with decreased communication with peers, playing videogames to cope, and poor sleep. First, healthy adolescents who maintained trust in peer relationships and often relied on communicating with their friends to cope with COVID-19 stress fared better than those who did not. Close peer relationships offer the forging of personal identities, support, and a sense of belonging and can buffer against negative impact of stress and development of mental health symptoms (31). Further, attachment in peer relationships (e.g., trust and communication) shows significant effects on positive mental health outcomes (32). Thus, the measures implemented to slow the spread of COVID-19 (i.e., social distancing, remote learning, canceled extracurricular activities, restrictions from in-person visits with friends and extended family) may have served to promote social isolation in adolescents, thereby adversely impacting psychological well-being.

Further, healthy adolescents who endorsed playing video games to cope exhibited greater increases in mental health symptoms. Time spent playing video games and the related problematic gaming behaviors (e.g., use of games to escape or relieve negative moods) have been associated with depression, anxiety, and physical health problems in adolescents (33). Although the consequences of the pandemic (e.g., sheltering in place) may have allowed for greater video game usage in healthy adolescents already engaging with video games, it may be the case that playing video games also interfered with the use of adaptive coping strategies, such as exercise, healthy sleep, and meaningful social interactions, thus contributing to negative moods. Finally, although the majority of healthy adolescents endorsed using sleep as a coping strategy, those who did not, evidenced greater increases in depression and anxiety. Previous research supports the role of poor sleep in development and maintenance of affective disturbances in adolescents (34). Taken together, these findings suggest that prioritizing peer connections and healthy sleep, while limiting time spent playing video games, may have promoted adaptive coping and served a protective role against negative moods.

Finally, rates of family conflict remained the same in both groups across time. Adolescents within the ELS group endorsed higher family conflict than healthy adolescents, but neither group showed any significant changes. Healthy adolescents also reported higher levels of trust and communication with both parents than did ELS adolescents, although these differences remained stable throughout the pandemic for both groups. Therefore, we conclude that changes in family dynamics did not impact the observed changes in mental health outcomes in healthy adolescents. While several studies have demonstrated or warned against increases in family violence following the onset of the pandemic, especially for adolescents already at risk for abuse (35–38), these findings were not present in our sample.

## Limitations

Although the current study is among the first to examine changes in mental health symptoms using the longitudinal approach (i.e., with pre-pandemic measurements available), there are limitations to consider. First, larger studies with a greater number of predictors (i.e., sex, age, coping strategies) are needed to further examine the impact the pandemic has had on adolescent mental health. Second, generalizability may be limited. The experience of the COVID-19 pandemic thus far has been dependent upon the location and timeframe in which data is collected. For the present study, data was collected in a midwestern state near the beginning of phased reopenings and thus represents only a snapshot in time. Cases were relatively low when compared with the spikes the U.S. has observed in more recent months. Finally, a group of anxious or depressed adolescents without ELS exposure would help further delineate changes in mental health symptoms in response to COVID-19 and shed light on how different trauma profiles may impact symptom outcomes over time.

## Conclusions

We examined mental health symptoms among healthy adolescents and adolescents with histories of early life stress prior to and after the onset of the COVID-19 pandemic. We discovered increases in anxiety and depression symptoms in previously healthy adolescents, whereas ELS adolescents demonstrated no significant changes in symptoms. We further observed a number of factors that may have served a protective role during COVID-19, including peer trust and communication, engagement with positive activities, and quality sleep, while coping strategies such as playing video games may have served as a risk factor for negative mental health outcomes. Prevention and intervention efforts may be able to capitalize on these factors to improve outcomes among youth affected by large-scale stressors. Further, it is likely that the COVID-19 pandemic is exacerbating an increasingly upward trend in mental health disorders in adolescents and young adults, further pointing to the need for timely interventions to prevent public mental health crises.

## Data Availability Statement

The data that support the findings of this study are available from the corresponding author upon reasonable request.

## Ethics Statement

The studies involving human participants were reviewed and approved by Western Institutional Review Board. Written informed consent to participate in this study was provided by the participants' legal guardian/next of kin.

## Author Contributions

ZC contributed to the data collection, data analysis and interpretation, literature search, writing of the manuscript, and creation of tables and figures. KC and DD contributed to the data analysis and interpretation, literature search, and writing of the manuscript. EA contributed to the writing of the manuscript. MS, RA, and MP contributed to the study design and revisions to the manuscript. EW and JS contributed to the revisions of the manuscript. NK contributed to the study design, data analysis and interpretation, and writing of the manuscript. All authors contributed to the article and approved the submitted version.

## Conflict of Interest

The authors declare that the research was conducted in the absence of any commercial or financial relationships that could be construed as a potential conflict of interest.

## Footnotes

^1^Alvis L, Shook N, Oosterhoff B. Adolescents' prosocial experiences during the covid-19 pandemic: associations with mental health and community attachments. (unpublished manuscript).
