# Supplementary material for: The Impact of COVID-19 on Adolescent Mental Health: Preliminary Findings From a Longitudinal Sample of Healthy and At-Risk Adolescents
Source: Front Pediatr. 2021 Jun 8;9:622608. doi: 10.3389/fped.2021.622608 (PMC8217763; doi:10.3389/fped.2021.622608)
Supplement: Supplementary file 1 [file Data_Sheet_1.DOCX]

**Supplementary Information**

**Title:** The impact of COVID-19 on adolescent mental health: Preliminary findings from a longitudinal sample of healthy and at-risk adolescents

**Authors:** Zsofia P. Cohen, BS^1^, Kelly T. Cosgrove, MA^1,2^, Danielle C. Deville, MA^1,2^, Elisabeth Akeman, MS^1^, Manpreet K. Singh, MD, MS^3^, Evan White, PhD^1^, Jennifer L. Stewart, PhD^1,4^, Robin L. Aupperle^1,4^, PhD, Martin P. Paulus, MD^1^, and Namik Kirlic^1^, PhD

**Affiliations:** ^1^Laureate Institute for Brain Research, Tulsa, OK; ^2^Department of Psychology, University of Tulsa, Tulsa, Oklahoma; ^3^Department of Psychiatry and Behavioral Sciences, Stanford University, Palo Alto, California; ^4^School of Community Medicine, University of Tulsa, Tulsa, Oklahoma

**Corresponding Author:** Namik Kirlic, PhD, Laureate Institute for Brain Research,

6655 South Yale Ave. Tulsa, OK 74136. nkirlic@laureateinstitute.org; Phone: (918) 502-5747

**Supplementary Materials**

1. Supplementary tables
   1. Table S1. COVID-19 Pandemic-related experiences.
   2. Table S2. Correlations in healthy controls.
   3. Table S3. T-tests between changes in depression, anxiety, and coping skills used in healthy controls.

| **Table S1. COVID-19 Pandemic-related experiences.** | | | | | | | |
| --- | --- | --- | --- | --- | --- | --- | --- |
| COVID-19 Characteristics | ELS (n=9) | | HC (n=15) | | Total (n=24) | | Group Differences |
|  | N | % | N | % | N | % | χ2 |
| COVID Symptoms |  |  |  |  |  |  | 2.70 |
| Cough | 2 | 22% | 0 | 0% | 2 | 8% |  |
| Fatigue | 0 | 0% | 2 | 13% | 2 | 8% |  |
| Fever | 1 | 11% | 1 | 7% | 2 | 8% |  |
| Loss of Taste or Smell | 0 | 0% | 0 | 0% | 0 | 0% |  |
| Shortness of Breath | 1 | 11% | 0 | 0% | 1 | 4% |  |
| Sore Throat | 1 | 11% | 0 | 0% | 1 | 4% |  |
| None | 6 | 67% | 13 | 87% | 19 | 79% |  |
| Family Diagnosis |  |  |  |  |  |  | 2.28 |
| Yes, Household member | 1 | 11% | 0 | 0% | 1 | 4% |  |
| Yes, Non-household member | 0 | 0% | 1 | 7% | 1 | 4% |  |
| No | 8 | 89% | 14 | 93% | 22 | 92% |  |
| Suspected Diagnosis |  |  |  |  |  |  | 2.28 |
| Positive Test | 1 | 11% | 0 | 0% | 1 | 4% |  |
| Possible Symptoms, No Diagnosis | 0 | 0% | 1 | 7% | 1 | 4% |  |
| Medical Diagnosis, No Test | 0 | 0% | 0 | 0% | 0 | 0% |  |
| Not Suspected | 9 | 100% | 15 | 100% | 24 | 100% |  |
| Outcomes due to COVID-19 for family members |  |  |  |  |  |  | 1.55 |
| Fallen Ill Physically | 0 | 0% | 0 | 0% | 0 | 0% |  |
| Hospitalized | 0 | 0% | 1 | 7% | 1 | 4% |  |
| Loss of Job | 1 | 11% | 2 | 13% | 3 | 13% |  |
| Passed Away | 0 | 0% | 0 | 0% | 0 | 0% |  |
| Reduced Ability to Earn Money | 1 | 11% | 5 | 33% | 6 | 25% |  |
| Self-Quarantine with Symptoms | 0 | 0% | 2 | 13% | 2 | 8% |  |
| Self-Quarantine without Symptoms | 2 | 22% | 3 | 20% | 5 | 21% |  |
| None | 6 | 67% | 8 | 53% | 14 | 58% |  |
| Exposure to someone with COVID-19 |  |  |  |  |  |  | 0.23 |
| Positive Test | 0 | 0% | 0 | 0% | 0 | 0% |  |
| Possible Symptoms, No Diagnosis | 2 | 22% | 1 | 7% | 3 | 13% |  |
| Medical Diagnosis, No Test | 0 | 0% | 0 | 0% | 0 | 0% |  |
| No | 7 | 78% | 14 | 93% | 21 | 88% |  |
| Household Essential Workers |  |  |  |  |  |  | 0.20 |
| Yes | 7 | 78% | 9 | 60% | 16 | 67% |  |
| No | 2 | 22% | 6 | 40% | 8 | 33% |  |
| Situations Applied to your Household |  |  |  |  |  |  | 2.79 |
| Local government encouraging people to stay home | 8 | 89% | 14 | 93% | 22 | 92% |  |
| Parent is a medical professional in quarantine | 3 | 33% | 2 | 13% | 5 | 21% |  |
| Stay-in-order by local government | 4 | 44% | 10 | 67% | 14 | 58% |  |
| Voluntary quarantine due to confirmed case | 0 | 0% | 3 | 20% | 3 | 13% |  |
| Voluntary quarantine due to fear of exposure | 5 | 56% | 8 | 53% | 13 | 54% |  |
| Activities your Family Stopped |  |  |  |  |  |  | 6.86 |
| Contact with Family Inside the Home | 1 | 11% | 1 | 7% | 2 | 8% |  |
| Contact with Family Outside the Home | 4 | 44% | 8 | 53% | 12 | 50% |  |
| Contact with Friends Indoors | 4 | 44% | 7 | 47% | 11 | 46% |  |
| Contact with Friends Outdoors | 3 | 33% | 2 | 13% | 5 | 21% |  |
| Family Travel | 6 | 67% | 9 | 60% | 15 | 63% |  |
| Family Activities in Outdoor Spaces | 2 | 22% | 5 | 33% | 7 | 29% |  |
| Family Activities in Public Spaces | 3 | 33% | 10 | 67% | 13 | 54% |  |
| Going to Restaurants or Stores | 3 | 33% | 12 | 80% | 15 | 63% |  |
| Indoor Exercise | 3 | 33% | 7 | 47% | 10 | 42% |  |
| In-Person Community Events | 8 | 89% | 9 | 60% | 17 | 71% |  |
| In-Person Religious Services | 7 | 78% | 9 | 60% | 16 | 67% |  |
| How are you coping with the stress or anxiety related to the COVID-19 outbreak? |  |  |  |  |  |  | 5.77 |
| Arts and Crafts | 5 | 56% | 4 | 27% | 9 | 38% |  |
| Board Games or Cards | 2 | 22% | 9 | 60% | 11 | 46% |  |
| Drinking Alcohol | 0 | 0% | 0 | 0% | 0 | 0% |  |
| Eating Comfort Foods | 7 | 78% | 9 | 60% | 16 | 67% |  |
| Eating Healthier | 2 | 22% | 2 | 13% | 4 | 17% |  |
| Exercising | 4 | 44% | 8 | 53% | 12 | 50% |  |
| Getting a Good Night's Sleep | 3 | 33% | 11 | 73% | 14 | 58% |  |
| Helping Others | 2 | 22% | 2 | 13% | 4 | 17% |  |
| Increased Self-Care Activities | 4 | 44% | 4 | 27% | 8 | 33% |  |
| Listening to Music | 8 | 89% | 13 | 87% | 21 | 88% |  |
| Meditation | 3 | 33% | 2 | 13% | 5 | 21% |  |
| More Family Activities | 2 | 22% | 8 | 53% | 10 | 42% |  |
| Not Skipping Prescribed Medications | 2 | 22% | 0 | 0% | 2 | 8% |  |
| Playing an Instrument | 4 | 44% | 4 | 27% | 8 | 33% |  |
| Playing Video Games | 4 | 44% | 12 | 80% | 16 | 67% |  |
| Prayer | 2 | 22% | 4 | 27% | 6 | 25% |  |
| Reading | 5 | 56% | 7 | 47% | 12 | 50% |  |
| Spending Time with Pets | 7 | 78% | 10 | 67% | 17 | 71% |  |
| Taking New Prescription Drugs | 0 | 0% | 0 | 0% | 0 | 0% |  |
| Taking Vitamins | 2 | 22% | 2 | 13% | 4 | 17% |  |
| Talking to Friends | 8 | 89% | 11 | 73% | 19 | 79% |  |
| Talking to Mental Health Professionals | 2 | 22% | 2 | 13% | 4 | 17% |  |
| Texting or Other Social Media | 8 | 89% | 10 | 67% | 18 | 75% |  |
| Using Marijuana | 0 | 0% | 2 | 13% | 2 | 8% |  |
| Using Other Recreational Drugs | 0 | 0% | 0 | 0% | 0 | 0% |  |
| Using Tobacco | 0 | 0% | 1 | 7% | 1 | 4% |  |
| Watching Movies | 9 | 100% | 8 | 53% | 17 | 71% |  |
| Writing | 2 | 22% | 2 | 13% | 4 | 17% |  |
| Perceived impact of COVID-19 | ELS (n=9) | | HC (n=15) | | Range | | Group Differences |
|  | Mean | SD | Mean | SD | ELS | HC | t |
| Cognitive Disruptions | 31.56 | 5.79 | 27.53 | 7.39 | 24 - 42 | 15 - 40 | 1.39 |
| Emotional Effects | 12.22 | 4.12 | 12.13 | 4.03 | 6 - 20 | 6 - 21 | 0.05 |
| Extent of Negative Emotions | 40.00 | 14.32 | 29.80 | 8.05 | 14 - 64 | 14 - 43 | 2.25** |
| Extent of Positive Emotions | 10.67 | 4.66 | 15.53 | 3.87 | 6 - 18 | 8 - 20 | -2.76** |
| Has the quality of the relationships between you and members of your family changed? | 2.67 | 0.87 | 3.33 | 0.90 | 2 - 5 | 2 - 4 | -1.78* |
| Has the quality of your relationships with your friends changed? | 3.22 | 0.83 | 3.20 | 0.68 | 3 - 5 | 3 - 4 | 0.07 |
| How often are you getting outside of your house for allowed shelter-in-place activities? | 2.56 | 1.24 | 2.53 | 1.64 | 1 - 5 | 1 - 5 | 0.03 |
| How often have you complied with social distancing or shelter-in place restrictions? | 3.56 | 0.88 | 3.80 | 1.21 | 2 - 5 | 1 - 5 | -0.53 |
| How stressful have the restrictions on leaving home been for you? | 2.11 | 0.78 | 2.40 | 1.35 | 1 - 3 | 1 - 5 | -0.58 |
| How worried have you been that someone in your house or extended family will get sick? | 2.00 | 0.87 | 2.07 | 1.28 | 1 - 3 | 1 - 5 | -0.14 |
| Level of concern related to impact of COVID19 pandemic | 40.22 | 9.27 | 34.80 | 12.46 | 24 - 59 | 18 - 58 | 1.13 |
| Negative Impact on Life | 3.00 | 1.22 | 2.93 | 1.33 | 1 - 5 | 1 - 5 | 0.12 |
| Positive Impact on Life | 1.89 | 0.93 | 2.53 | 1.13 | 1 - 3 | 1 - 5 | -1.44 |
| Stress regarding disruption of existing plans | 2.78 | 1.20 | 2.33 | 1.23 | 1 - 5 | 1 - 4 | 0.86 |
| Stress regarding uncertainty about the future | 2.78 | 1.56 | 2.13 | 1.13 | 1 - 5 | 1 - 4 | 1.17 |
| *** p < .01 ** p < .05 * p < .10  Abbreviations: ELS, early life stress; HC, healthy control. | | | | | | | |

| **Table S2.** Correlations in healthy controls (n=15) | | | | | | | | | | | |
| --- | --- | --- | --- | --- | --- | --- | --- | --- | --- | --- | --- |
|  | Soc  Connect | Soc  Needs | Change in PeerComm | Change in PeerAlien | Change in Peer Trust | Worry | Hopelessness | Concerns | Uncertainty | Disruptions | Restrictions |
| Change in Depression | 0.24 | -0.07 | -0.29 | -0.29 | -0.54** | -0.19 | 0.08 | 0.16 | 0.09 | 0 | 0 |
| Change in Anxiety | 0.07 | -0.14 | -0.47* | -0.3 | -0.64*** | 0.06 | 0.55** | 0.38 | 0.46* | 0.19 | 0.16 |
| Note. Correlations are examined in healthy controls only. Degrees of freedom was 13 for all correlations except for Social Connectedness, which had 8 degrees of freedom due to missing data.  *** p < .01 ** p < .05 * p < .10  Abbreviations: Soc Connect, Social Connectedness; Soc Needs, Social Needs Met; PeerComm, Peer Communication; PeerAlien, Peer Alienation; Worry, Worries about COVID-19; Hopelessness, Increased Feelings of Hopelessness; Concerns, Concerns about the Impact of COVID-19; Uncertainty, Stress from Uncertainty; Disruptions; Stress from Disruptions; Restrictions, Stress from Restrictions on Leaving Home. | | | | | | | | | | | |

| **Table S3**. T-tests between changes in depression, anxiety, and coping skills used in healthy controls. | | | | | | | | | | |
| --- | --- | --- | --- | --- | --- | --- | --- | --- | --- | --- |
| Coping Strategy | Change in Depression | | | | | Change in Anxiety | | | | |
|  | Endorsed | | Not Endorsed | | Group Differences | Endorsed | | Not Endorsed | | Group Differences |
|  | Mean | SD | Mean | SD | t | Mean | SD | Mean | SD | t |
| Arts and Crafts | 0.10 | 0.23 | 0.31 | 0.36 | -0.06 | 0.01 | 0.10 | 0.26 | 0.28 | -0.48 |
| Board or Card Games | 0.19 | 0.32 | 0.36 | 0.36 | -1.97* | 0.12 | 0.22 | 0.30 | 0.31 | -2.44** |
| Eating Comfort Foods | 0.19 | 0.34 | 0.35 | 0.34 | -1.99* | 0.18 | 0.33 | 0.20 | 0.16 | -2.74** |
| Exercising | 0.21 | 0.35 | 0.31 | 0.34 | -1.63 | 0.19 | 0.33 | 0.19 | 0.19 | -2.29** |
| Getting a Good Night's Sleep | 0.21 | 0.30 | 0.38 | 0.45 | -2.95** | 0.19 | 0.28 | 0.19 | 0.23 | -3.97*** |
| Increased Self-Care | 0.03 | 0.19 | 0.34 | 0.34 | -0.06 | -0.03 | 0.06 | 0.27 | 0.26 | -0.46 |
| More Family Activities | 0.11 | 0.24 | 0.42 | 0.37 | -1.46 | 0.12 | 0.21 | 0.27 | 0.31 | -2.05* |
| Playing Instrument | 0.14 | 0.17 | 0.30 | 0.38 | -0.07 | 0.08 | 0.18 | 0.23 | 0.29 | -0.51 |
| Playing Video Games | 0.27 | 0.37 | 0.19 | 0.16 | -4.18*** | 0.22 | 0.29 | 0.06 | 0.08 | -5.50*** |
| Prayer | 0.06 | 0.15 | 0.33 | 0.33 | -0.06 | 0.10 | 0.20 | 0.22 | 0.28 | -0.51 |
| Reading | 0.18 | 0.30 | 0.33 | 0.37 | -1.21 | 0.12 | 0.18 | 0.25 | 0.32 | -1.68 |
| Spending Time with a Pet | 0.34 | 0.32 | 0.22 | 0.35 | -2.49** | 0.17 | 0.29 | 0.23 | 0.22 | -3.17** |
| Talking with Friends | 0.21 | 0.33 | 0.38 | 0.36 | -2.94** | 0.17 | 0.28 | 0.25 | 0.23 | -3.76** |
| Texting and Other Social Media | 0.15 | 0.29 | 0.46 | 0.35 | -2.26** | 0.16 | 0.30 | 0.25 | 0.20 | -3.13** |
| Watching Movies | 0.17 | 0.22 | 0.36 | 0.42 | -1.55 | 0.08 | 0.20 | 0.32 | 0.28 | -1.95* |
| Note. The number of healthy subjects endorsing the use of each coping skill is shown in Table S1. Analyses include coping skills that were endorsed by 20% to 80% of healthy control subjects.  *** p < .01 ** p < .05 * p < .10 | | | | | | | | | | |
